# Coronary collateralization shows sex and racial-ethnic differences in obstructive artery disease patients

**DOI:** 10.1371/journal.pone.0183836

**Published:** 2017-10-10

**Authors:** Zhi Liu, Margaret A. Pericak-Vance, Pascal Goldschmidt-Clermont, David Seo, Liyong Wang, Tatjana Rundek, Gary W. Beecham

**Affiliations:** 1 John P. Hussman Institute for Human Genomics, University of Miami, Miami, Florida, United States of America; 2 Division of Cardiology, Miller School of Medicine, University of Miami, Miami, Florida, United States of America; 3 Department of Neurology, Miller School of Medicine, University of Miami, Miami, Florida, United States of America; Medical University Innsbruck, AUSTRIA

## Abstract

**Background:**

Coronary collateral circulation protects cardiac tissues from myocardial infarction damage and decreases sudden cardiac death. So far, it is unclear how coronary collateralization varies by race-ethnicity groups and by sex.

**Methods:**

We assessed 868 patients with obstructive CAD. Patients were assessed for collateral grades based on Rentrop grading system, as well as other covariates. DNA samples were genotyped using the Affymetrix 6.0 genotyping array. To evaluate genetic contributions to collaterals, we performed admixture mapping using logistic regression with estimated local and global ancestry.

**Results:**

Overall, 53% of participants had collaterals. We found difference between sex and racial-ethnic groups. Men had higher rates of collaterals than women (P-value = 0.000175). White Hispanics/Latinos showed overall higher rates of collaterals than African Americans and non-Hispanic Whites (59%, 50% and 48%, respectively, P-value = 0.017), and especially higher rates in grade 1 and grade 3 collateralization than the other two populations (P-value = 0.0257). Admixture mapping showed Native American ancestry was associated with the presence of collaterals at a region on chromosome 17 (chr17:35,243,142-41,251,931, β = 0.55, P-value = 0.000127). African ancestry also showed association with collaterals at a different region on chromosome 17 (chr17: 32,266,966-34,463,323, β = 0.38, P-value = 0.00072).

**Conclusions:**

In our study, collateralization showed sex and racial-ethnic differences in obstructive CAD patients. We identified two regions on chromosome 17 that were likely to harbor genetic variations that influenced collateralization.

## Introduction

Coronary artery disease (CAD) is the leading cause of death worldwide, with atherosclerosis being the major etiology. Currently, much is known about CAD pathogenesis and risk factors [1], such as age, gender, smoking, serum cholesterol, metabolism rate and hypertension [2]. While this knowledge is useful in predicting CAD, it is often insufficient to adequately predict clinical outcomes after cardiac events, such as myocardial infarction (MI). As such, other factors may play a critical role in clinical outcomes. One such factor is the presence of a collateral circulation. Collateral circulation is a natural bypass process, whereby vessels are extended or expanded to supply blood flow to cardiac muscle that may otherwise be lacking oxygen [3]. Collateralization is protective and likely reduces mortality [4, 5], and has been shown to have a protective effect among those with CAD [5, 6]. Indeed, the extent of collateralization significantly determines the severity of MI in acute coronary occlusion–the better developed collateral circulation, the less severe of the MI outcomes [7].

The extent of the collateral circulation varies significantly among healthy individuals as a function of both genetic background [8, 9] and vascular risk factors such as aging, diabetes and smoking [10]. In animal studies, genetic heterogeneity leads to different levels of collateral formation, suggesting genetic predisposition [8, 11, 12]. Array-based expression profiling in mice showed 783 out of 12000 genes were differently expressed between femoral artery ligation group and control group. Among those differentially expressed genes, several angiogenesis related genes were upregulated in cases, such as monocyte chemoattractant protein-1, placental growth factor and cysteine-rich protein-61 [13, 14].

The rates of collateralization between men and women remain unclear. Some studies showed that there was no significant effect of sex on collateral vessel development [15]. However, others showed that in acute coronary syndrome men tended to develop greater collateral circulation than women [16]. The opposite was reported in another study, where collaterals were more frequent in women than in men with multivessel disease [17]. While race-ethnicity groups clearly show prevalence differences in CAD, it is unclear if rates of collateralization differ across race-ethnicity groups.

To investigate factors influencing coronary collateralization, we identified 868 obstructive CAD patients from a cardiac catheterization lab. These patients were investigated for collateral circulation, to assess the impact of sex, race-ethnicity, and other risk factors with collateralization. Additionally, we performed admixture mapping to identify regions of the genome that may contribute to differential collateralization across the race-ethnic groups.

## Methods

### Sample assessment

This study features a retrospective design approved by the University of Miami institutional review board (IRB). All subjects initially presented to the cardiac catheterization laboratories at the University of Miami Medical Center or Jackson Memorial Hospital for coronary angiography from year 2007 to 2012. The initial dataset included 2023 consecutive patients from the Miami Cardiovascular Registry. Patients who had non-obstructive CAD were excluded due to the different prevalence of collateralization between obstructive and non-obstructive CAD patients. 868 patients with obstructive coronary artery disease (over 75% stenosis presented in at least one of the 16 coronary artery segments designated from the American Heart Association model [18]). Blood was drawn prior to the start of the cardiac catheterization. All patients were consented in written.

### Phenotyping

Coronary collaterals were defined as an anastomotic connection without an intervening capillary bed between portions of the same coronary artery and between different coronary arteries [6]. Collateral levels were assessed based on Rentrop collateral grading system [19] by experienced cardiologists: grade 0 = none; grade 1 = filling of side branches only; grade 2 = partial filling of the main epicardial recipient artery; grade 3 = complete filling of the main epicardial recipient artery. The maximum collateral grade at any given location was used as phenotype in our analyses. We also assessed a presence/absence measure of collateralization that did not distinguish between grades. Additional variables considered included age, sex, race-ethnicity, blood pressure, and current comorbidities such as diabetes, smoking history, presence/absence of hypertension or cholesterol lowering medications and reason for procedures.

Based on occlusions occurred in the number of three major coronary arteries (left anterior descending, left circumflex, and right coronary artery), patients were diagnosed to have one (1VD), two (2VD) or three (3VD) vessel diseases, and three-vessel disease was the severest type among all three. Patients with occlusions presented in the left main coronary artery were diagnosed to have left main disease.

### Genotyping and quality control

DNA was extracted from patient blood samples and genotyping was performed on the Affymetrix 6.0 GeneChip array using established protocols. We retained 838,221 SNPs after appropriate SNP quality control (QC) with PLINK [20]: SNP call rate ≥ 0.95, minor allele frequency (MAF) > 0.01, Hardy-Weinberg equilibrium (HWE) P-value > 1 x 10^−5^ and autosomal chromosome. Sample QC included sample call rate ≥ 0.95, removal of sex mismatch and related individuals. The EIGENSTRAT software [21] was used to assess the genetic ancestry of the samples (S1 Fig).

### Statistical analysis

Analysis of variance (ANOVA) test with two-tail hypothesis were performed on continuous traits (e.g., age) to compare the difference of means between four collateral classification groups. Chi-square tests were performed to compare categorical trait differences (such as sex, race-ethnicity, etc.) between classification groups.

Ordinal logistic regression was performed in R with collateral classification as the trait. Among covariates recorded, we selected those that showed moderate to significant differences in collateralization to be included into the statistical model: collateral grade = β_0_ + β_1_ sex + β_2_ smoke + β_3_ diabetes + β_4_ race-ethnicity + ε.

To perform admixture mapping, we estimated local ancestry, which included several steps listing below.

#### Haplotype phasing

We phased our genotyping data with SHAPEIT [22] to determine the possible haplotypes for each individual, based on reference populations of European, African and Asian from 1000 Genome Project [23]. We first built up a phasing graph of our population, which contained all possible haplotypes, and then we extracted 100 pairs of possible haplotypes from the built graph. The 100 pairs of haplotypes well represented the distribution of possible haplotypes and captured phasing uncertainty due to lack of family trio data.

#### Local ancestry inference

We inferred local ancestry of the 100 possible pairs of haplotypes using LAMP-LD/LAMP-ANC [24]. Reference populations were European and African from 1000 Genome Project, and Native American from Human Genome Diversity Project (HGDP) [25] because these ancestry populations well represented the ancestor of our study population. After ancestry inference, we counted ancestry states, averaged across haplotype pairs, and calculated the percentage of ancestry for each locus to be included in ancestry mapping.

#### Global ancestry inference

We averaged local ancestry across the genome to calculate global ancestry—they were highly correlated with principal component method estimated global ancestry.

#### Admixture mapping

Logistic regression model was used to estimate the effect of global ancestry and the difference between global and local ancestry on collaterals. After controlling for global ancestry, we calculated the association between the phenotype and the difference between global and local ancestry at each locus. We extracted β and P-value for each ancestry component to analyze the effect of ancestry on collateral phenotype.

$$\text{logit}\;\left(\text{Y}\right)=\beta_{0}\prime +\beta_{1}\prime {\;\text{G}}_{\text{AA}}+\beta_{2}\prime {\;\text{G}}_{\text{NA}}+\beta_{3}\prime \;\left({\text{G}}_{\text{AA}}-{\text{L}}_{\text{AA}}\right)+\beta_{4}\prime \;\left({\text{G}}_{\text{NA}}-{\text{L}}_{\text{NA}}\right)+\beta_{5}\prime {\;\text{age}+\beta }_{6}\prime \;\text{sex}+\varepsilon$$

Where, Y: 0 = no-collaterals; 1 = collaterals. G_AA_: global African ancestry. G_NA_: global Native American ancestry. L_AA_: local African ancestry. L_NA_: local Native American ancestry.

#### Permutation correction

To correct for multiple testing, we performed a permutation test. The phenotype was permuted 2000 times; for each permutation P-values were calculated for the local ancestry components as described above, and we recorded the minimum P-value across the entire genome. We then estimated the number of tests by dividing 0.05 to the 5^th^ percentile distribution of the minimum P-value. The P-value at the 5^th^ percentile represents the 0.05 P-value corrected for multiple testing.

To test genetic association within the two regions identified by admixture mapping, SNPs were tested for association with collateralization using PLINK [20]. The statistical model for association test was: logit (Y) = β_0_ + β_1_ genotype + β_2_ eigenvector1 + β_3_ eigenvector2 + β_4_ eigenvector5 + β_5_ age + β_6_ sex + ε. Eigenvectors 1, 2, 5, age and sex were selected as covariates since these covariates were significantly correlated with collateral phenotype (P-value < 0.05). The R package *leaps* [26] was used to assess covariates. The number of independent SNPs in each region was assessed using a permutation test, as described above.

## Results

Among the 868 subjects with obstructive CAD, 404 (47%) had no collateralization (grade 0); 142 (16%) had maximum grade 1 collateralization at any location; 205 (24%) had maximum grade 2 collateralization at any locations and 117 (13%) had maximum grade 3 collateralization at any location. There was no difference in the average age among patients with different grades of collateralization (P-value = 0.068). Men were more likely to have collateralization than women in all collateral grades, with chi-square test P-value = 0.000175 (Table 1). Smoking was also significantly associated with collateralization, with smokers tending to have higher rates of collateralization than non-smokers (chi-square P-value = 0.00223) (Table 1). Diabetes, systolic and diastolic blood pressure (BP), hypertension, anti-hypertensive medicine and lipid lowering medicine usage, did not show statistically significant differences among patients with different grade of collateralization (Table 1).

**Table 1:** Population demographics and clinical characteristics based on collateral grades.

	Grade 0	Grade 1	Grade 2	Grade 3	P-value
**Age (SD)**	72 (13)	72 (11.6)	71 (12)	69 (11)	0.068
**Sex (Female)**	32%	27%	20%	14%	0.000175
**DBP (SD)**	76 (13)	76 (13)	77 (14)	76 (13)	0.889
**SBP (SD)**	141 (23)	140 (24)	139 (25)	136 (25)	0.291
**Diabetes**	43%	53%	41%	51%	0.068
**Smoking**	51%	50%	63%	66%	0.00223
**Hypertension**	73%	73%	71%	70%	0.909
**Anti-hypertensive meds**	86%	89%	86%	85%	0.706
**Anti-cholesterol meds**	30%	33%	36%	38%	0.401

DBP: diastolic blood pressure in mmHg; SBP: systolic blood pressure in mmHg.

To better understand the relationship between sex, smoking, and collateralization, we also tested the association between sex and smoking status to see if they were confounded. Indeed, men were more likely to smoke as compared to women (62% vs 38% smokers, respectively, p < 0.0001). We included covariates that showed moderate to significant differences in collateralization into ordinal logistic regression model (sex, smoking status, diabetes and race-ethnicity), and the result indicated that sex is statistically associated with collateralization while including smoking, diabetes and race-ethnicity in the model (β = 0.59, P-value = 0.000162). Smoking status also showed significant association with collateralization (β = 0.38, P-value = 0.00523) while controlling for sex, diabetes and race-ethnicity. However, when we included an interaction term of sex and smoking into the regression model, neither smoking nor the interaction term was significant (P-value > 0.05).

Collateralization was also significantly associated with the adverse prognosis of CAD. Since all patients in this study had obstructive CAD, we investigated the relationship between the extent and severity of CAD (1VD, 2VD and 3VD) and collateral grades. There were 21 patients with missing CAD extent and severity information that were excluded from this analysis. Among all patients with non-missing CAD extent and severity information, we found that there was a relationship between the extent and severity of CAD and collateral grades (P-value = 3.29E-22, df = 6). Patients with severer forms of CAD were more likely to develop collateralization than patients with less severe forms of CAD (Table 2). When stratifying patients by the presence/absence of collateralization, we had similar results as compared to classifying patients by collateral grades (S3 Table). In addition, we investigated the relationship between collateralization and the presence/absence of left main disease, and found that collateralization was also associated with the presence/absence of left main disease (chi-square P-value = 0.013). Patients with left main disease were more likely to develop collateralization than patients without left main disease (S2 Table).

**Table 2:** The relationship between the extent and severity of CAD and collateralization grades.

	Grade 0	Grade 1	Grade 2	Grade 3	Total
**1VD**	207 (66%)	33 (11%)	48 (15%)	26 (8%)	314 (100%)
**2VD**	118 (49%)	39 (16%)	46 (19%)	39 (16%)	242 (100%)
**3VD**	69 (24%)	65 (22%)	106 (36%)	51 (18%)	291 (100%)
**Total**	394 (47%)	137 (16%)	200 (24%)	116 (14%)	847 (100%)

Chi-square P-value = 3.29E-22, df = 6. 1VD: one-vessel disease; 2VD: two-vessel disease; 3VD: three-vessel disease.

Using principal components analysis of the genotypes, we grouped individuals into Caucasians (247 individuals), Hispanics (487 individuals) and African Americans (92 individuals) (Table 3). There were 42 individuals with unclear race/ethnicities based on principal components that were excluded from this analysis. Among the 826 individuals with race-ethnicity determined, the different race-ethnicity groups showed different rates of collateralization: overall, 59% of Hispanic patients had collaterals as compared to 50% in African Americans and 48% in Caucasians (S1 Table). Hispanic patients tended to have higher rates of collateralization in grade 1 and grade 3 as compared to the other two populations (chi-square P-value = 0.0257) (Table 3). In ordinal logistic regression model, after controlling for sex, diabetes and smoking status, race-ethnicity comparisons also showed statistically significant difference in collaterals between Hispanics and Caucasians (β = -0.39, P-value = 0.00845). However, Hispanics and African Americans comparison was not statistically significant (β = -0.23, P-value = 0.276). We also classified collateralization by presence/absence, and results were similar as compared to classify collaterals by grade (S1 and S2 Tables).

**Table 3:** EIGENSTRAT defined race-ethnicity differences in collateralization within obstructive CAD patients.

	Grade 0	Grade 1	Grade 2	Grade 3	Total
**AA**	47 (51%)	13 (14%)	23 (25%)	9 (10%)	92 (100%)
**CAUC**	129 (52%)	30 (12%)	61(25%)	27 (11%)	247 (100%)
**HISP**	202 (41%)	94 (19%)	114 (23%)	77 (16%)	487 (100%)
**Total**	378 (47%)	137 (16%)	198 (24%)	113 (13%)	826 (100%)

Chi-square P-value = 2.57E-02, df = 6. AA: African Americans; CAUC: Caucasians; HISP: white Hispanics/Latinos.

**Fig 1:**
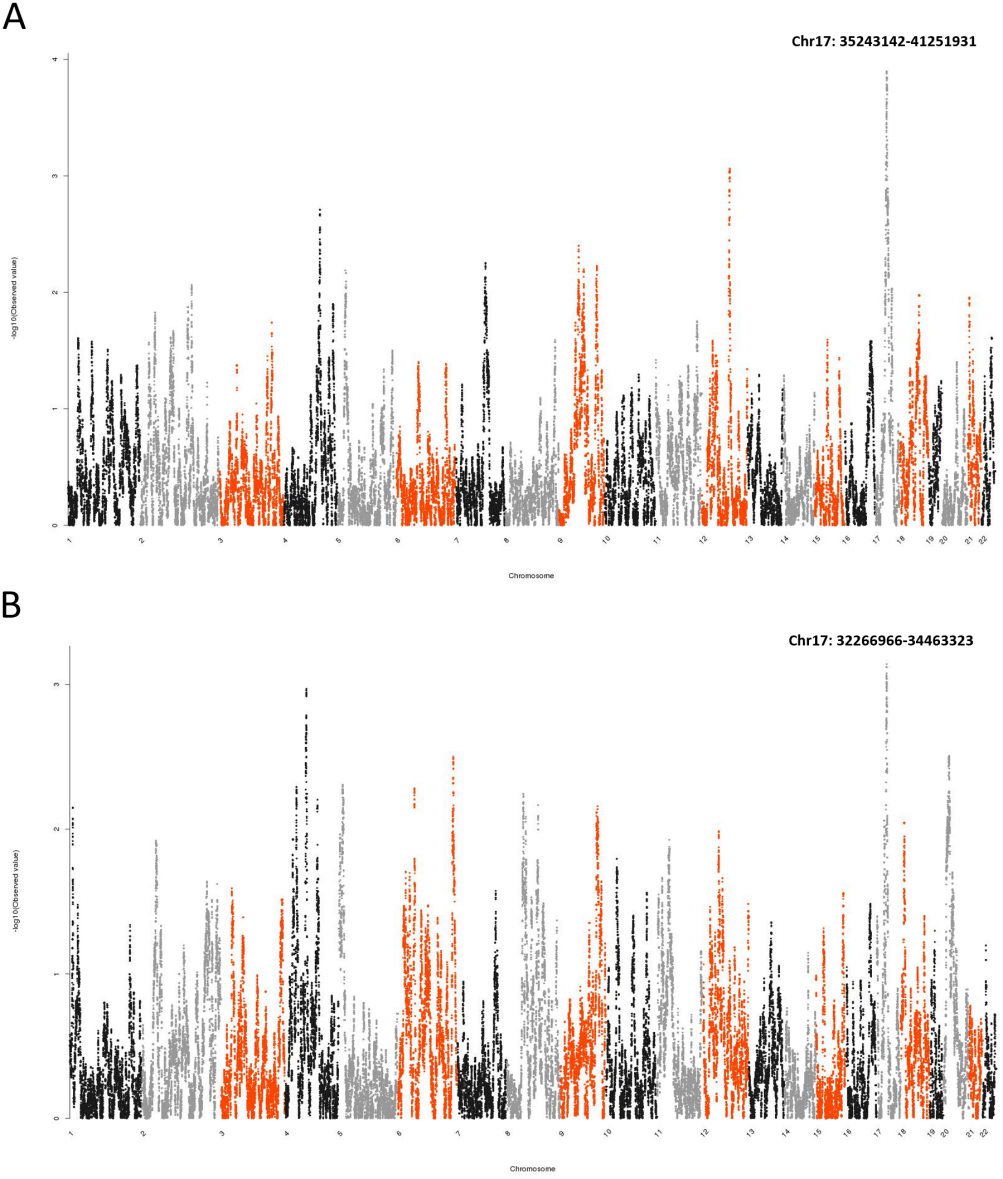
Manhattan plots showed associations between collateralization and local ancestry. (A) local Native American ancestry; (B) local African ancestry in admixture mapping. The peak regions of local Native American ancestry and local African ancestry were both located on chromosome 17. X-axis indicated chromosomes 1 to 22. Y-axis indicated −log10 of local Native American/African ancestry P-values.

**Fig 2:**
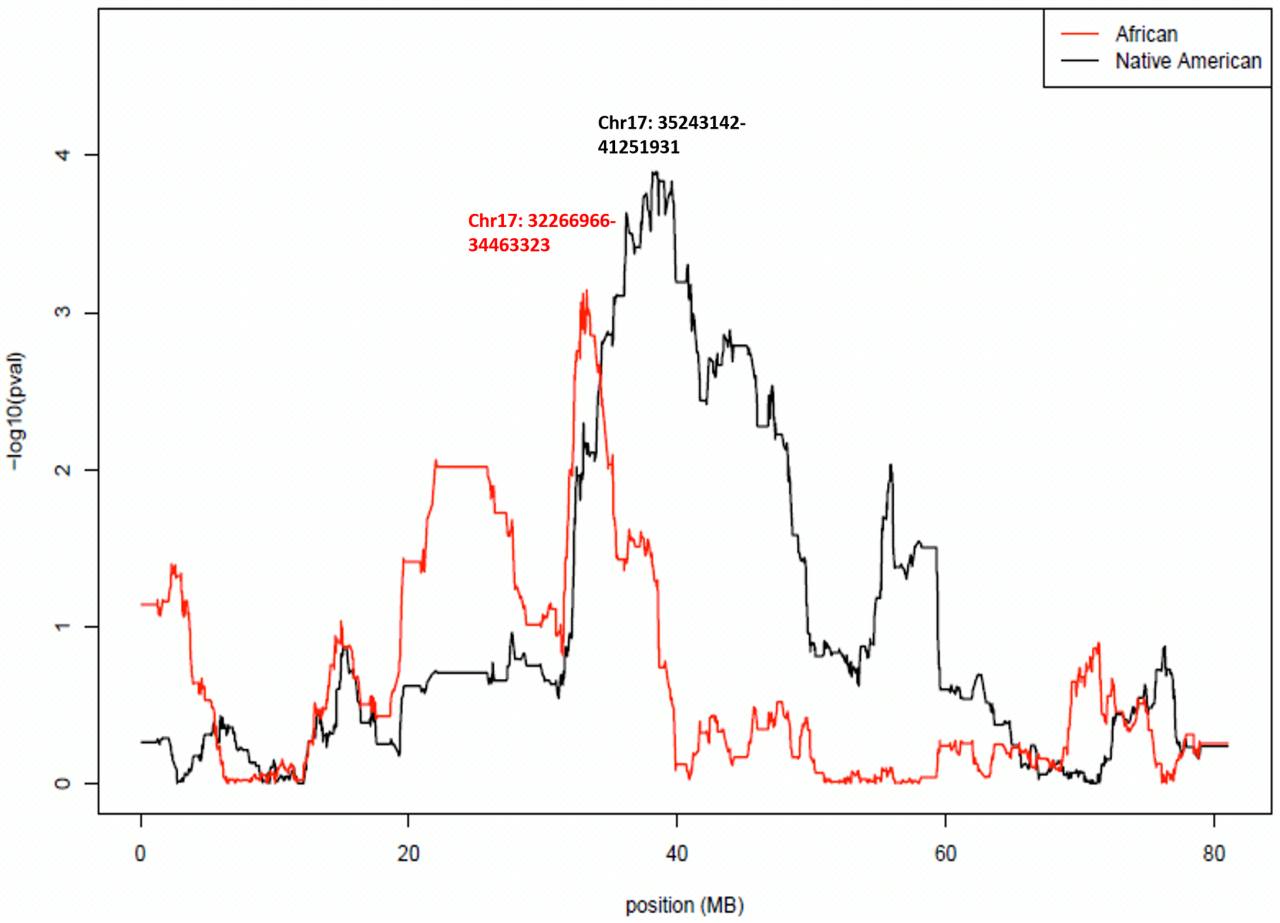
Chromosome 17 regional plot showed peak association between local African/Native American ancestries and collateralization. Local Native American ancestry was highly associated with the presence of collaterals at a region on chromosome 17 (35,243,142-41,251,931 (hg19), min P-value = 0.000127, −log10 (P-value) = 3.90). Local African ancestry also showed association with collaterals at a different region on chromosome 17 (32,266,966-34,463,323 (hg19), min P-value = 0.00072, -log10 (P-value) = 3.14). X-axis represented the base pair location on chromosome 17; Y-axis represented −log10 P-values.

We performed admixture mapping on 868 subjects of obstructive CAD patients, with the presence/absence of collateralization as the endpoint phenotype. Four covariates were tested for their association with collateral phenotype: global African ancestry, global Native American ancestry; the difference between global and local ancestry for African ancestry and Native American ancestry (delta African and delta Native American). Global African and Native American ancestry was not associated with collateral phenotype (P-value > 0.05). Local Native American ancestry was highly associated with the presence of collaterals at a region on chromosome 17 (35,243,142-41,251,931, hg19), β = 0.55, min P-value = 0.000127) (Figs 1A and 2). Local African ancestry also showed association with collaterals at a different region on chromosome 17 (32,266,966-34,463,323, hg19), β = 0.38, min P-value = 0.00072) (Figs 1B and 2). These P-values did not survive a multiple-testing correction based on our permutation test (1931 effective tests in the local African Ancestry analysis, P-value threshold = 2.59E-05; 1543 effective tests in the Native American ancestry analysis, P-value threshold = 3.249E-05).

Within the two identified regions on chromosome 17, we performed association tests between SNPs and the collateral phenotype. The SNP rs8071313 showed strong evidence of association (P-value = 0.0002394) that approached the regionally significant P-value cut-off (P-value = 0.000111, corresponding to 452 independent SNPs tested). This SNP is intronic to RAD51 paralog D (*RAD51D*), and near Notchless protein homolog 1 (*NLE1*), suggesting these genetic markers may play a role in collateralization.

## Discussion

### Sex differences in collateral formation

Coronary collateralization is a natural neovascularization process that provides additional blood supply to bypass highly stenotic region. It is a protective mechanism against CAD. In our dataset, men had significantly higher rates of collaterals as compared to women. In the past two decades, more women than men died of CAD [27], which can be attributed to the fact that women undergo less aggressive treatment or are under-representative in clinical trial studies, but also can partially be due to significantly lower rate of collaterals as compared to men. As collateralization is a protective mechanism after a cardiovascular event such as MI, men are more likely to have better outcomes following MI than women because of the additional collateral vessels circulate oxygen-enriched blood for heart muscle to recover and therefore prevent further damage.

### Effect of smoking

In our data, smokers had higher rate of collaterals as compared to non-smokers, and smoking status showed significant association with collaterals after accounting for sex, diabetes and race-ethnicity differences. While smoking status was significantly associated when testing by itself, neither it, nor its interaction term was significant when including an interaction term between sex and smoking. Since sex and smoking were highly correlated, smoking could be confounded with sex and showed a difference in collateralization, due to more men smokers than women smokers in our data set. However, it is also possible that smoking status can truly affect collateralization through inducing hypoxia, which triggers collateralization, but the effect of smoking was not as strong as sex to show a significant association when including the interaction term. One study previously showed that smoking was positively associated with the presence of collateralization, while pack-years of smoking was not related [28]. We further investigated the association between pack-years and collateralization in our dataset, and concluded that pack-years were not statistically significantly associated with either the presence/absence or the grade of collateralization.

### Candidate genes within these two regions on chromosome 17

Within these two regions identified on chromosome 17, where African and Native American ancestries were associated with collateralization, we found several candidate genes. Region chr17:35,243,142-41,251,931 (hg19) harbors several interesting genes where Native American ancestry was associated with collateralization in our data. For example, human junction plakoglobin (*JUP*) has a function of stimulating VE-cadherin in endothelial cells [29]. Titin cap protein (*TCAP*) specifically expressed in heart and skeletal muscle. It is responsible for muscle assembly regulation, and its mutation has been shown to affect cardiomyopathy [30]. There are also genes that has been known to have significant roles in other biological functions, such as breast cancer 1 (*BRCA1*), a well-studied breast cancer gene and signal transducer and activator of transcription 3 (*STAT3*), an intermediate component in signaling pathways for cytokines and growth factors such as interleukin-6 (*IL-6*) and vascular endothelial growth factor (*VEGF*). In region chr17:32,266,966-34,463,323 (hg19) where African ancestry was highly associated with collateralization, a candidate gene is mitochondrial rRNA methyltransferase 1 (*MRM1*), which has been shown to be positively associated with blood pressure determination, cholesterol and low-density lipoprotein (LDL) [31]. Regional association test suggested that *NLE1* and *RAD51D* may influence collateralization. *NLE1* plays a role in regulating Notch activity, which involves in cell-cell communication and cell fate determination [32]. *RAD51D* has been shown to play a role in DNA repair by catalyzing homologous recombination [33].

### Race-ethnicity differences in collateral formation

We categorized our population based on eigenvalues from principal component (PC) analysis instead of self-report for several reasons: first, In Miami, population admixture is more complicated than elsewhere in the US, therefore it can be difficult for individuals to trace back admixture history of their ancestors and know exactly their ancestry category. Second, individuals may not understand differences between each ancestry categories or self-identify their ancestry category based on vague knowledge. Therefore, to eliminate ambiguity, we think PC based ancestry is more reliable than self-identified ancestry.

Hispanics is an admixed population composed of different proportions of European, African and Native American ancestries. Our dataset showed that Hispanics had the highest rate of collaterals as compared to African Americans and Non-Hispanic Whites. Both Native American and African ancestry showed association with collateralization on chromosome 17, at two loci located close to each other. African ancestry showed association with collateralization with a slightly lower association signal than Native American ancestry. Since both Native American and African ancestry have a positive effect on collateral, we think that these two ancestries contribute to a higher rate of collaterals in Hispanic populations. Hispanics have the highest rate of collateral may due to the highest proportion of Native American than Non-Hispanic Whites and African Americans. It is likely that due to the presence of African Ancestry at this specific locus, African Americans have higher rate of collateral than Non-Hispanic Whites, because Non-Hispanic Whites usually do not have either African or Native American at these loci.

### Other factors influencing collateralization

We investigated the relationship between collateralization and the extent and severity of CAD, and found that the grade of collateralization was associated with adverse prognosis of CAD. In addition, patients with left main disease were more likely to develop collateralization as compared to those without left main disease. Previous studies supported our conclusions that the severity of coronary artery stenosis positively influenced collateralization [19, 34].

Anti-cholesterol medicine was not statistically significantly associated with collateralization in our dataset. Previous studies suggested hypercholesterolemia was associated with the presence of coronary collaterals [35], and was observed more frequent in patients with greater angiographic apparent collateral grade [36]. In these studies, hypercholesterolemia was identified by cholesterol level measured in the blood stream, but in our study, hypercholesterolemia was evaluated by the usage of anti-cholesterol medicine, rather than directly measuring cholesterol level from the blood stream. These two distinct ways of evaluating hypercholesterolemia may explain different outcomes of the effect of cholesterol on collateralization.

In our study, diabetes showed only nominal association with collateralization (P-value = 0.068). Previous studies indicated patients with diabetes develop poorer collaterals than patients without diabetes [37]. Another study suggested that there were no differences between diabetes and non-diabetes stable CAD patients in quantitatively measured coronary collateral flow index [38]. We stratified our diabetic patients by the presence/absence of insulin treatment regardless of brands, dosage and the way to take the medicine, and tested the relationship between the presence/absence of insulin treatment and collateralization among diabetic patients. No relationship was found between diabetic insulin treatment status and collateral grades or the presence/absence of collateralization.

### Limitation of the study

In healthy individuals, lower resting heart rate is associated with increased collateralization [39]. For patients with chronic stable CAD, heart rate reduction also appears to increase coronary collateral growth [40]. All our patients had obstructive CAD, but we did not have heart rate information or ivabradine usage by these patients on file.

Inflammation factors may also induce collateral growth, such as the pro-inflammatory agent lipopolysaccharide (LPS) increases the degree and speed of collateralization [41]. Therefore, some autoimmune diseases may potentially affect collateralization. Since the inflammation data were incomplete in our dataset, we could not exclude those patients who may have autoimmune and inflammation diseases. However, among the records we had, the prevalence of inflammation/autoimmune disease in our dataset was very low (<2%), indicating that they are not likely to have a large impact on our results.

Due to small sample size, we had limited statistical power, which might explain that although our findings were interesting, they did not reach genome-wide significance after correcting for multiple testing.

## Conclusions

In summary, we assessed sex and race-ethnicity differences in collateralization. We showed that men have higher rate of collateralization than women, which may in part explains why women have poorer outcomes after MI than men. Different race-ethnicity groups have different rates of collateralization, which may contribute our knowledges to racial disparities in MI outcomes. Finally, we identified two regions on chromosome 17 that are likely to harbor genetic variations that influence collateralization. This study provides us insights to understand disparities of CAD patients from different sex and racial/ethnical backgrounds, and help us understand the genetics components for coronary collateralization.

## Supporting information

S1 Fig Principal components determined race-ethnicity for 826 individuals who participated in this study. Red: Hispanics; green: Caucasians; blue: African Americans.
(DOCX)

S1 Table EIGENSTRAT defined race-ethnicity differences in presence/absence of collateralization within obstructive CAD patients. (DOCX)

S2 Table Population demographics and clinical characteristics based on the presence/absence of collateralization. (DOCX)

S3 Table The relationship between the presence/absence of collateralization and the extent and severity of CAD. (DOCX)

S1 Phenotype File Phenotypes for study participants. (XLSX)

S1 Covariate File Covariates for study participants. (XLSX)

S1 BED File Genotype file for participants with collateralization in PLINK bed format. (BED)

S2 BED File Genotype file for participants without collateralization in PLINK bed format. (BED)

S1 BIM File Genotype file for participants with collateralization in PLINK bim format. (BIM)

S2 BIM File Genotype file for participants without collateralization in PLINK bim format. (BIM)

S1 FAM File Genotype file for participants with collateralization in PLINK fam format. (FAM)

S2 FAM File Genotype file for participants without collateralization in PLINK fam format. (FAM)
