# Supplementary material for: Coronary collateralization shows sex and racial-ethnic differences in obstructive artery disease patients
Source: PLoS One. 2017 Oct 10;12(10):e0183836. doi: 10.1371/journal.pone.0183836 (PMC5634541; doi:10.1371/journal.pone.0183836)

**S1 Fig. Principal components determined race-ethnicity for 826 individuals who participated in this study.** Red: Hispanics; green: Caucasians; blue: African Americans.


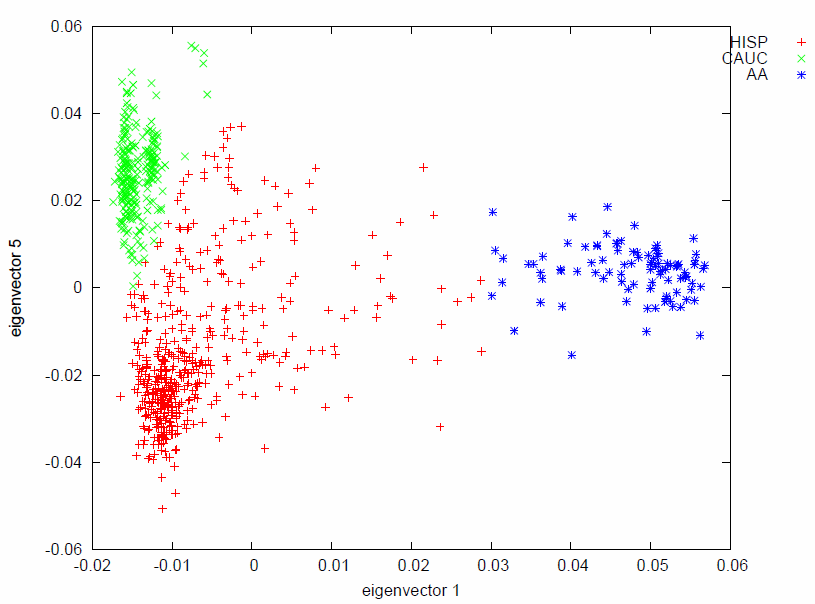

Supplement: S1 Fig — Red: Hispanics; green: Caucasians; blue: African Americans. (DOCX) [file pone.0183836.s001.docx]
