# Supplementary material for: Coronary collateralization shows sex and racial-ethnic differences in obstructive artery disease patients
Source: PLoS One. 2017 Oct 10;12(10):e0183836. doi: 10.1371/journal.pone.0183836 (PMC5634541; doi:10.1371/journal.pone.0183836)
Supplement: S1 Table — (DOCX) [file pone.0183836.s002.docx]

**S1 Table. EIGENSTRAT defined race-ethnicity differences in presence/absence of collateralization within obstructive CAD patients.**

|  | **Collaterals** | **No Collaterals** | **Total** |
| --- | --- | --- | --- |
| AA | 47 (50%) | 47 (50%) | 94 (100%) |
| CAUC | 120 (48%) | 129 (52%) | 249 (100%) |
| HISP | 289 (59%) | 204 (41%) | 493 (100%) |
| **Total** | **456 (55%)** | **380 (45%)** | **836 *(100%)** |

*The total sample size was 836 when we evaluated collateralization by the presence/absence as compared to 826 when we categorized collateralization by Rentrop classification. Chi-square P-value = 0.017; df = 2. AA: African Americans; CAUC: Caucasians; HISP: white Hispanics/Latinos.
