# Supplementary material for: Coronary collateralization shows sex and racial-ethnic differences in obstructive artery disease patients
Source: PLoS One. 2017 Oct 10;12(10):e0183836. doi: 10.1371/journal.pone.0183836 (PMC5634541; doi:10.1371/journal.pone.0183836)
Supplement: S2 Table — (DOCX) [file pone.0183836.s003.docx]

**S2 Table. Population demographics and clinical characteristics based on the presence/absence of collateralization.**

|  | **No Collaterals** | **Collaterals** | **P-Value** |
| --- | --- | --- | --- |
| Age (sd) | 72(13) | 71 (12) | 0.248 |
| Women | 32% | 21% | 1.4E-04 |
| Diabetes | 43% | 48% | 0.211 |
| SBP in mmHg (sd) | 141(23) | 138(25) | 0.099 |
| DBP in mmHg (sd) | 76(13) | 76(13) | 0.557 |
| Anti-cholesterol meds | 31% | 35% | 0.135 |
| Smoking | 52% | 60% | 0.016 |
| Anti-hypertensive meds | 86% | 87% | 0.742 |
| Hypertension | 73% | 71% | 0.610 |
| Left main disease | 12% | 18% | 0.013 |

Note: the total sample size was 879 when we evaluated collateralization by the presence/absence as compared to 868 when we categorized collateralization by Rentrop classificatioin.
