# Supplementary material for: Coronary collateralization shows sex and racial-ethnic differences in obstructive artery disease patients
Source: PLoS One. 2017 Oct 10;12(10):e0183836. doi: 10.1371/journal.pone.0183836 (PMC5634541; doi:10.1371/journal.pone.0183836)
Supplement: S3 Table — (DOCX) [file pone.0183836.s004.docx]

**S3 Table. The relationship between the presence/absence of collateralization and the extent and severity of CAD.**

|  | **No Collaterals** | **Collaterals** | **Total** |  |  |
| --- | --- | --- | --- | --- | --- |
| 1VD | 207 (66%) | 107 (34%) | 314 (100%) |  |  |
| 2VD | 118 (49%) | 124 (51%) | 242 (100%) |  |  |
| 3VD | 69 (24%) | 222 (76%) | 291 (100%) |  |  |
| Total | 394 (47%) | 453 (53%) | 847 (100%) |  |  |
| Chi-square P-value = 2.30E-24, df =2. 1VD: one-vessel disease; 2VD: two-vessel disease; 3VD: three-vessel disease. | | | | | |
